# 1880. Detection of Infectious SARS-CoV-2 in Specimens with High CT Values Is More Common for Omicron than for Delta Variants

**DOI:** 10.1093/ofid/ofac492.1507

**Published:** 2022-12-15

**Authors:** Michel Tassetto, Miguel Garcia-Knight, Khamal Anglin, Dan Kelly, Scott Lu, Jesus Pineda-Ramirez, Sharon Saydah, Melissa Briggs-Hagen, Amethyst Zhang, Ruth Diaz Sanchez, Kevin Donohue, Mariela Romero, Michael J Peluso, Jeffrey Martin, Raul Andino, Claire Midgley

**Affiliations:** UCSF, San Francisco, California; UCSF, San Francisco, California; UCSF, San Francisco, California; UCSF, San Francisco, California; UCSF, San Francisco, California; UCSF, San Francisco, California; Centers for Disease Control and Prevention, Atlanta, Georgia; Centers for Disease Control and Prevention, Atlanta, Georgia; UCSF, San Francisco, California; UCSF, San Francisco, California; UCSF, San Francisco, California; UCSF, San Francisco, California; University of California San Francisco, San Francisco, California; UCSF, San Francisco, California; UCSF, San Francisco, California; Centers for Disease Control and Prevention, Atlanta, Georgia

## Abstract

**Background:**

Although not validated, cycle threshold (Ct) values from real-time (r)RT-PCR are sometimes used as a proxy for infectiousness to inform public health decision-making. A better understanding of variant-specific viral dynamics, including RNA and infectious virus relationships, is needed to clarify implications for diagnostics and transmission.

**Methods:**

Non-hospitalized SARS-CoV-2-infected individuals were recruited ≤ 5 days post-onset and self-collected nasal swabs daily for two weeks. Sequencing was used to determine variant, an in-house quantitative rRT-PCR targeting *N* gene was used to produce Ct values and determine RNA load, and cytopathic effect was used to assess the presence or absence of infectious virus (binary outcome). We used a Ct threshold of 30 to define high-Ct (Ct > 30) or low-Ct (Ct ≤ 30) specimens and assessed the percentage of RNA-positive specimens that had infectious virus; variant-specific percentages were compared by Χ^2^ test.

**Results:**

We included 113 and 200 RNA-positive specimens from 18 and 28 Omicron- and Delta-infected participants, respectively; timing of RNA-positive specimen collection was similar in both groups (median = 8d post-onset). Maximum observed RNA levels occurred at median of 5 days post-onset for both variants but were lower for participants with Omicron vs Delta [mean RNA copies/mL = 10^5.2^ vs 10^7.9^]. Despite lower RNA levels, infectious virus was frequently detected for both variants [Omicron: median duration = 4.5d; Delta: median = 6d; *p* = 0.13]. Omicron specimens with infectious virus had higher Cts vs Delta specimens [mean Ct = 29.9 vs 23.2, *p* < 0.001]. In high-Ct specimens (Ct > 30; **Table**), the percentage of specimens with infectious virus was typically higher for Omicron vs Delta, and was significantly higher in adults [27.3% vs 9.5%]. In low-Ct specimens (Ct ≤ 30), the percentage with infectious virus was similar or higher for Omicron vs Delta, and was significantly higher in children [87.5% vs 53.8%] and in those unvaccinated [94.1% vs 47.4%].

**Figure:**
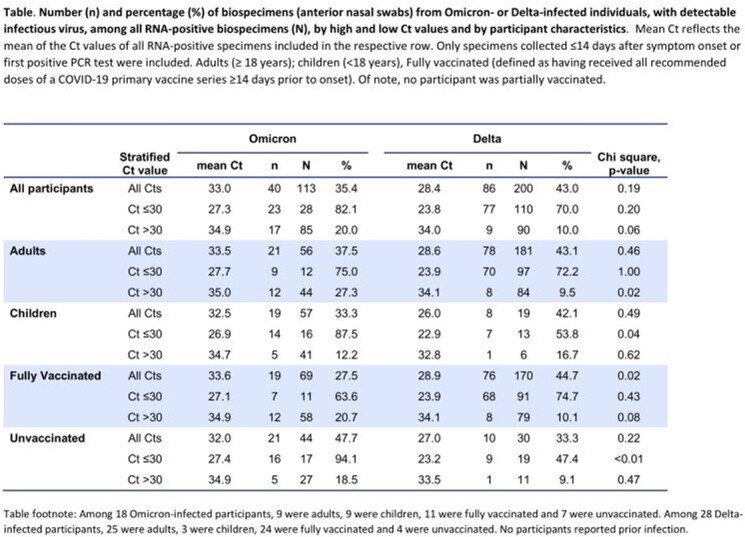

**Conclusion:**

CDC does not recommend the use of Ct values as a proxy for infectiousness. These data further highlight that Ct values may not provide a reliable or consistent proxy for infectiousness across variants.

**Disclosures:**

**All Authors**: No reported disclosures.

